# Single-molecule long-read sequencing facilitates shrimp transcriptome research

**DOI:** 10.1038/s41598-018-35066-3

**Published:** 2018-11-16

**Authors:** Digang Zeng, Xiuli Chen, Jinxia Peng, Chunling Yang, Min Peng, Weilin Zhu, Daxiang Xie, Pingping He, Pinyuan Wei, Yong Lin, Yongzhen Zhao, Xiaohan Chen

**Affiliations:** Guangxi Key Laboratory of Aquatic Genetic Breeding and Healthy Aquaculture, Guangxi Academy of Fisheries Sciences, Nanning, Guangxi P.R. China

## Abstract

Although shrimp are of great economic importance, few full-length shrimp transcriptomes are available. Here, we used Pacific Biosciences single-molecule real-time (SMRT) long-read sequencing technology to generate transcripts from the Pacific white shrimp (*Litopenaeus vannamei*). We obtained 322,600 full-length non-chimeric reads, from which we generated 51,367 high-quality unique full-length transcripts. We corrected errors in the SMRT sequences by comparison with Illumina-produced short reads. We successfully annotated 81.72% of all unique SMRT transcripts against the NCBI non-redundant database, 58.63% against Swiss-Prot, 45.38% against Gene Ontology, 32.57% against Clusters of Orthologous Groups of proteins (COG), and 47.83% against Kyoto Encyclopedia of Genes and Genomes (KEGG) databases. Across all transcripts, we identified 3,958 long non-coding RNAs (lncRNAs) and 80,650 simple sequence repeats (SSRs). Our study provides a rich set of full-length cDNA sequences for *L. vannamei*, which will greatly facilitate shrimp transcriptome research.

## Introduction

Whole-transcriptome analysis is of growing importance for animal biology research. However, whole-transcriptome analyses are ineffective without high quality transcript sequences^1^. Recently, second-generation sequencing (SGS) technologies, such as the Illumina Genome Analyzer, the Roche 454 pyrosequencing platform, and the ABI Solid platform, have facilitated the construction of transcriptome resources for many organisms^2,3^.

Shrimp are economically- and nutritionally-important crustaceans^4^. Several transcriptome studies in shrimp have been performed using SGS^5^, and many expressed sequence tags (ESTs) have been obtained^6^. However, the construction of transcriptomic sequences using SGS generally requires the assembly of short RNA-seq reads, and without a high-quality genome sequence available as a reference transcriptomic sequences may be misassembled due to reads transcribed from very similar members of multigene families or from highly repetitive regions^7^. In shrimp, the danger of misassembly may be even greater, as ~80% of the shrimp genome has been estimated to consist of repetitive elements^8^. Another limitation of SGS is that these technologies generally do not produce full-length transcripts, which are fundamental to studies of structural and functional genomics^9–11^. In addition, gene annotations and transcriptional characterizations of full-length transcripts are more accurate than those of transcript tags assembled from short RNA-sequencing reads^7^. Finally, alternative splicing, alternative polyadenylation, homologous genes, and superfamily genes are more easily identified based on full-length transcripts^12–15^.

Single-molecule real-time (SMRT) sequencing, a third-generation sequencing (TGS) technique recently developed by Pacific Biosciences (PacBio), allows direct sequencing of full-length, single-molecule cDNA sequences with a read length of up to 20 kb^9,11,16^. Using PacBio SMRT sequencing, intact RNA molecules can be sequenced without the need for fragmentation or post-sequencing assembly^9^. Thus, full-length transcripts can be constructed using SMRT sequencing.

The Pacific white shrimp (*Litopenaeus vannamei*) is the most extensively cultured crustacean species in the world, owing to its fast growth and strong disease resistance^17,18^. In this study, we used SMRT sequencing to construct the *L. vannamei* transcriptome. This is the first shrimp transcriptome constructed with SMRT.

## Results

### SMRT sequencing, quality filtering, and error correction

**Figure 1:**
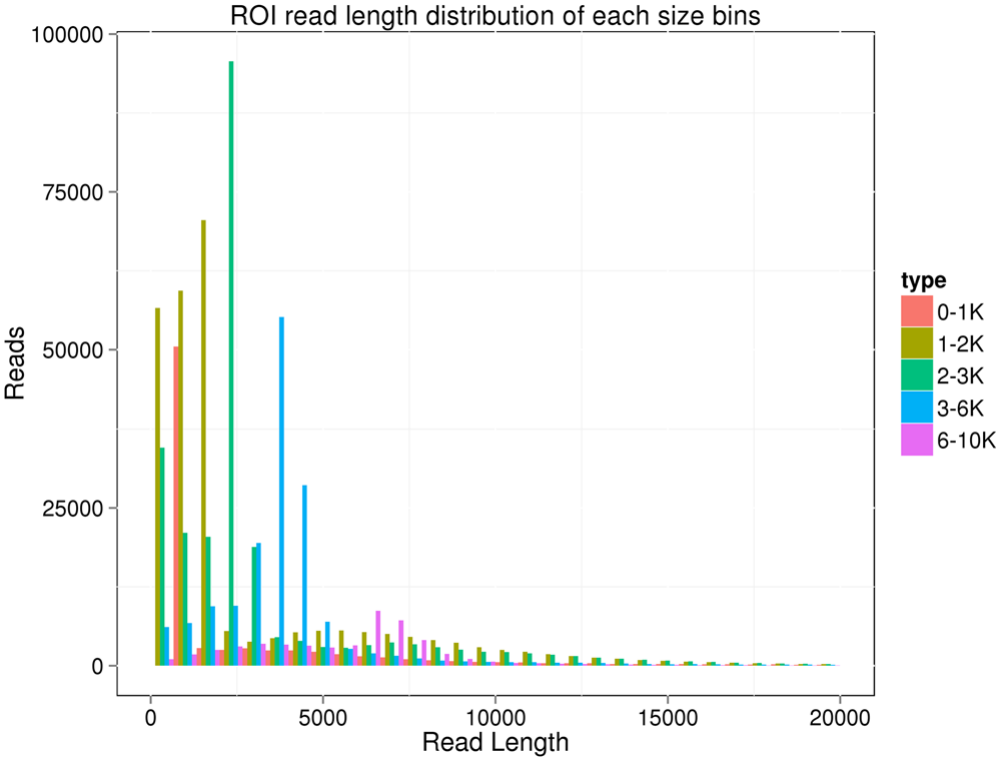
ROI read length distribution. Different colors represent different SMRT sequencing libraries with different cDNA insert size ranges.

We used RNA extracted from six tissues (hepatopancreas, gills, heart, intestine, muscle, and stomach), collected and pooled from six *L. vannamei*, to constructed five cDNA libraries, each including cDNA inserts of approximately the same size: <1 kb, 1–2 kb, 2–3 kb, 3–6 kb, and >6 kb. We generated 1,307,853 polymerase reads (30.9 gigabases) across all five libraries. After removing adaptor sequences, low-quality sequences, and short sequences (<50 bp), 12,920,542 sub-reads remained. The mean sequence lengths for five cDNA libraries were 789 bp (<1 kb); 1,438 bp (1–2 kb); 2,304 bp (2–3 kb); 3,766 bp (3–6 kb); and 6,834 bp (>6 kb). We obtained 828,618 ROIs across all five cDNA libraries; the average lengths of the ROIs across the cDNA libraries were 2,018 bp, 2,968 bp, 3,340 bp, 4,235 bp, and 5,913 bp, respectively (Fig. 1). Of the 828,618 ROIs, 322,600 (38.93%) were identified as full-length non-chimeric (FLNC) reads.

We performed Illumina library construction and sequencing in parallel to correct the 322,600 FLNC reads. Using Illumina, ~148 million paired-end reads were sequenced, from which ~132 million clean reads were generated after adaptor sequence trimming and low-quality read filtering. We used Proovread^19^ to correct the FLNC reads based on the Illumina short reads. Proovread indicated that 124,201 FLNC reads (38.50%) contained at least one erroneous inner and/or terminal fragment; these fragments were corrected. We then used iterative clustering for error correction (IEC) to obtain 51,367 unique corrected SMRT transcripts.

To further test the completeness of our transcriptome, we used the Benchmarking Universal Single-Copy Orthologs (BUSCO) pipeline^20^ to compare our *L. vannamei* transcriptome to 1,066 conserved arthropod genes. This analysis indicated that 81.0% of the *L. vannamei* transcriptome (863 genes) encoded complete proteins. Of these genes, 34.3% (366 genes) were complete single-copy BUSCOs, 46.6% (497 genes) were complete duplicated BUSCOs, 3.1% (33 genes) were fragmented BUSCO archetypes, and 16.0% (170 genes) were missing BUSCOs entirely.

### Functional annotation of transcripts

**Figure 2:**
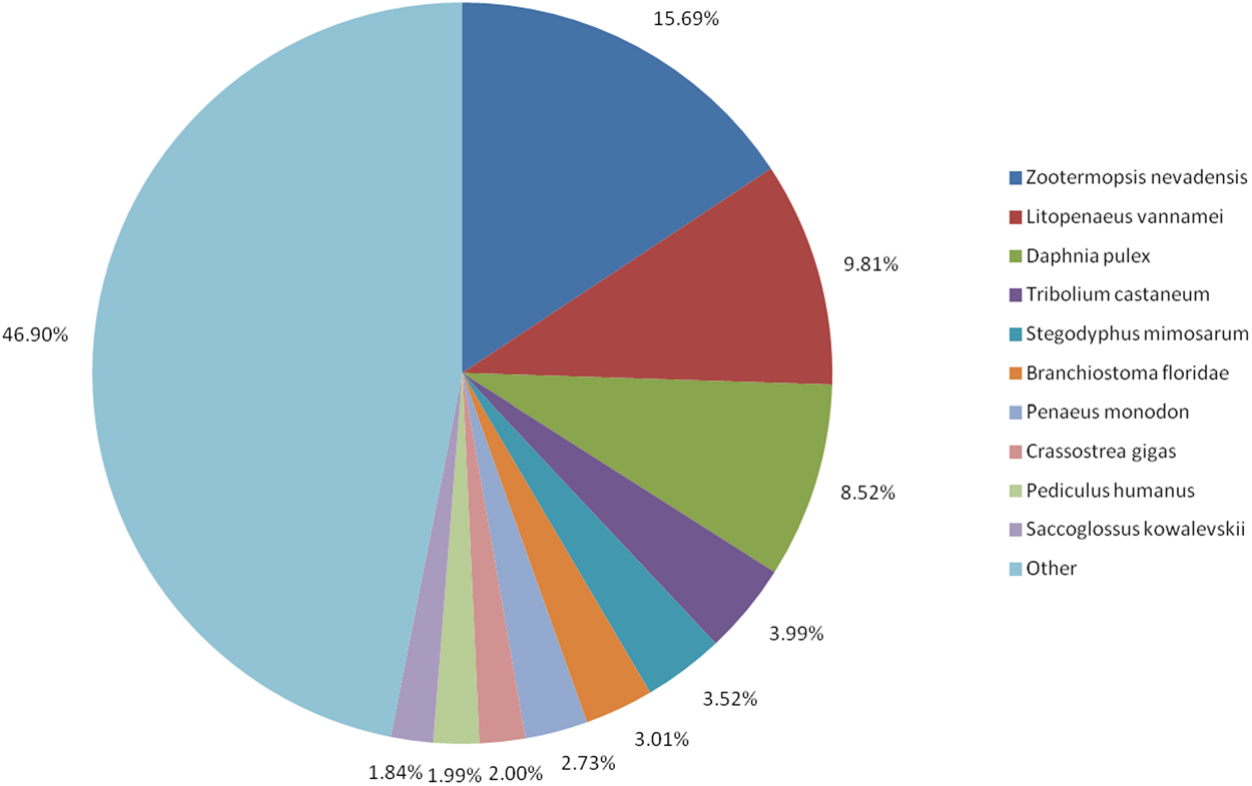
Percentage of *L. vannamei* transcripts with BlastX hits in various species. Transcripts were searched against the NCBI non-redundant protein database, using BlastX with the E-value cutoff set to <10^−5^. Only species with matches for >1.8% of the *L. vannamei* transcripts are shown; species matching fewer than 1.8% of all transcripts are classed as ‘Other’.

Of the 51,367 unique SMRT transcripts, we identified significant matches in the NCBI non-redundant (Nr) protein database for 41,975 (81.72%; E-value ≤ 10^−5^). Of the species with matches for >1.8% of all *L. vannamei* transcripts, 15.69% of the hits were from the termite *Zootermopsis nevadensis*, 9.81% were from *L. vannamei*, and 8.52% were from the crustacean *Daphnia pulex* (8.52%; Fig. 2).

**Figure 3:**
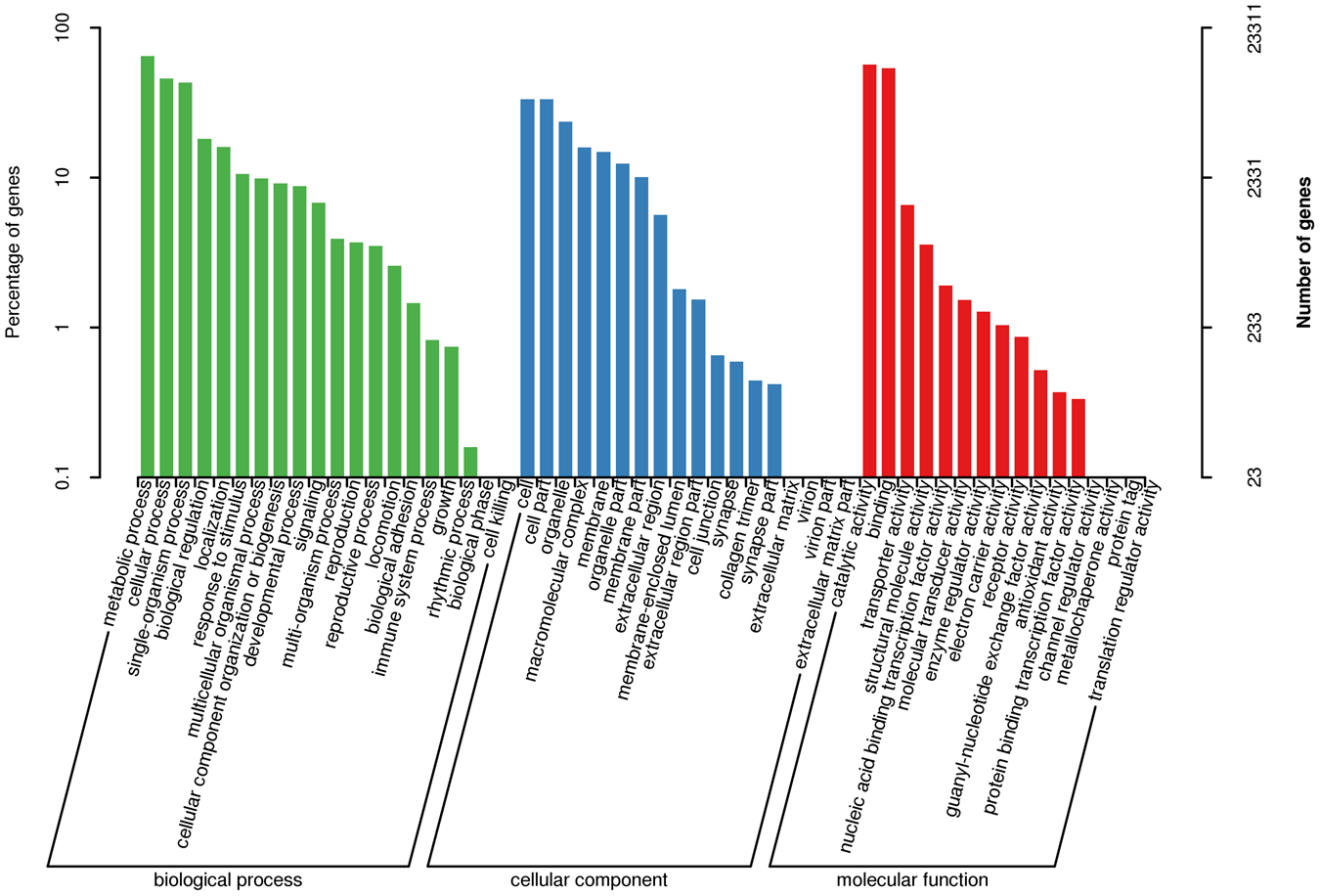
GO classification of the putative functions of the unique transcripts of *L. vannamei*.

Our gene ontology (GO) analysis indicated that 9910 of the unique transcripts (42.51%) were enriched in biological processes, 8129 (34.87%) were enriched in molecular functions, and 5272 (22.62%) were enriched in cellular components (Fig. 3). We also identified matches to our unique transcripts in the Swiss-Prot^21^, Clusters of Orthologous Groups of proteins (COG)^22^, and Kyoto Encyclopedia of Genes and Genomes (KEGG)^23^ databases: 30,117 transcripts matched an entry in Swiss-Prot (58.63%), 16,732 transcripts matched an entry in COG (32.57%), and 24,569 transcripts matched an entry in KEGG (47.83%). The functional annotation of all unique transcripts are listed in Supplementary Table 1.

**Figure 4:**
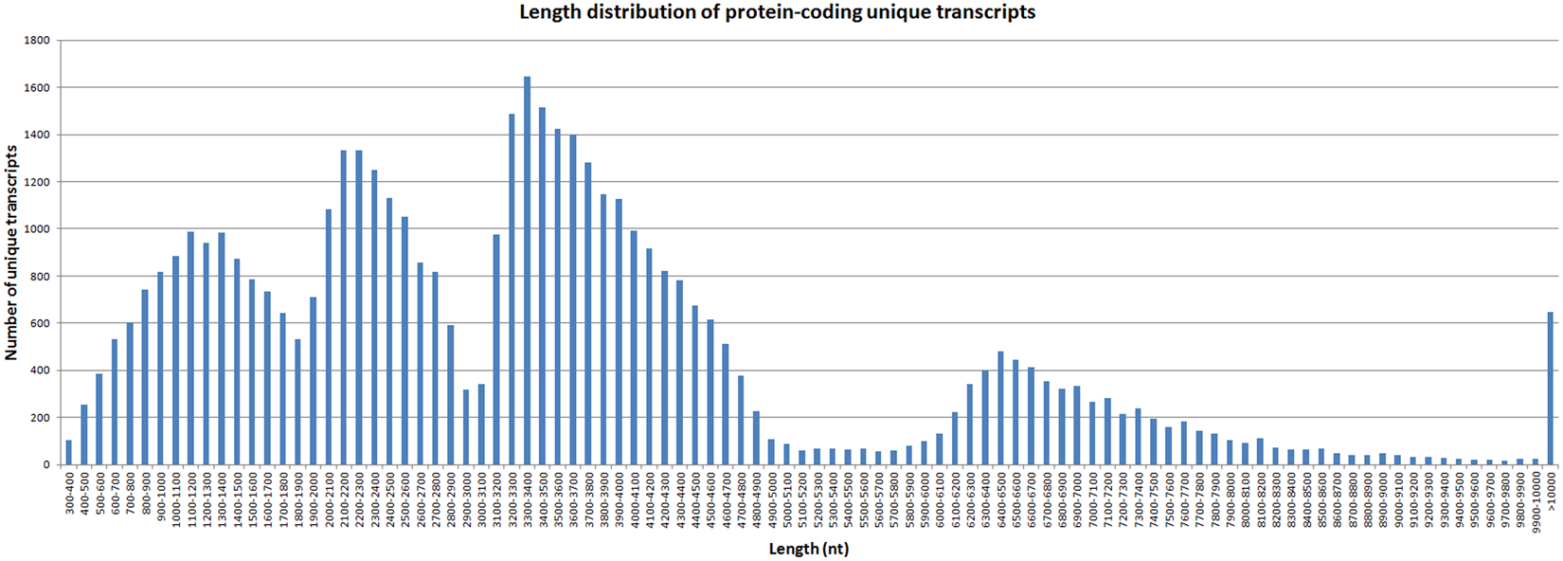
Lengths of candidate protein-coding RNAs.

To further identify the protein coding potential of unique transcripts, we predicted ORFs within all unique transcripts. In total, 47,260 unique transcripts were found having the protein coding potential, with an average length of 3,493 bp. The length distribution indicated that most protein-coding unique transcripts were distributed in length from 300 bp to 1,0000 bp, and there were more than 600 transcripts with a length >10,000 bp. (Fig. 4).

### Identification of long non-coding RNAs (lncRNAs)

**Figure 5:**
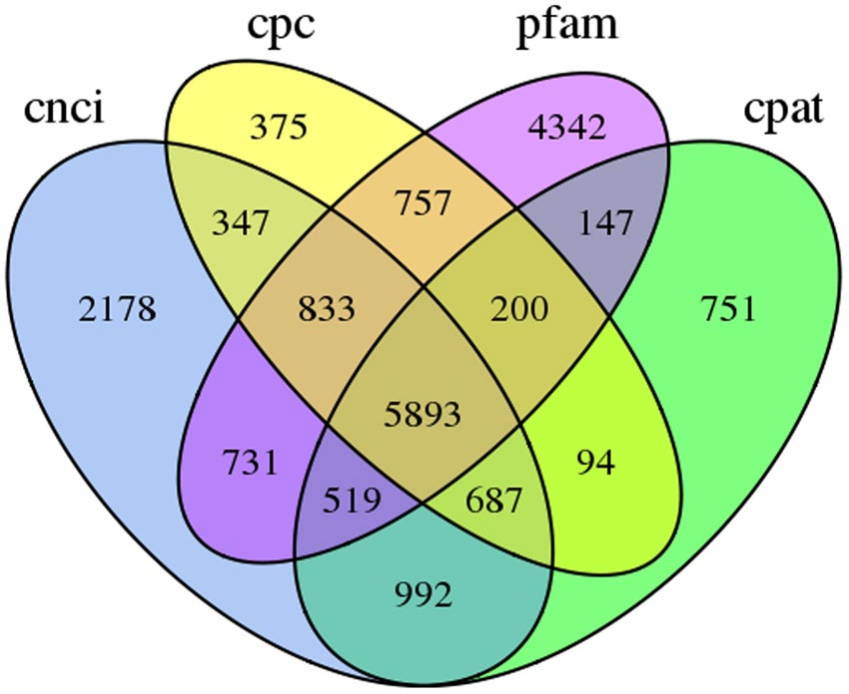
Candidate lncRNAs identified using CPC^24^, CNCI^25^, CPAT^26^, and Pfam^27^. Un-overlapping areas indicate the number of lncRNAs identified by the single tool; overlapping areas indicate the total number of lncRNAs identified by the several tools.

**Figure 6:**
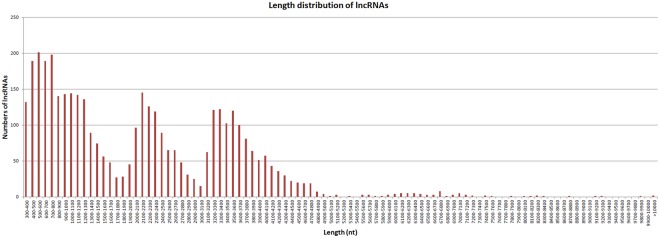
Lengths of candidate lncRNAs.

We used four tools to identity unique transcripts without protein coding potential (i.e., lncRNAs): the Coding Potential Calculator (CPC)^24^ identified 375 lncRNAs, the Coding-Non-Coding Index (CNCI)^25^ identified 2,178 lncRNAs, the Coding Potential Assessment Tool (CPAT)^26^ identified 751 lncRNAs, and Pfam^27^ identified 4,342 lncRNAs. In total, 5893 unique transcripts were identified as lncRNAs by at least one tool (Fig. 5). After candidate lncRNAs with EMBOSS-predicted ORFs > 100 bp were removed, 3,958 lncRNAs remained. The average length of these lncRNAs was 2,111 bp, with most lncRNAs ranging in length from 300 bp to 4,800 bp (Fig. 6).

### Identification of simple sequence repeats (SSRs)

SSRs are repetitive sequence motifs approximately 1–6 bp long^28^. We searched for SSRs in the 50,688 unique *L. vannamei* transcripts longer than 500 bp. We identified 80,650 SSRs across all tested transcripts, with 17,222 (33.98%) unique transcripts containing more than one SSR. Most of the SSRs identified were mono-nucleotide repeats (50.81%), followed by the di-nucleotide repeats (27.55%), tri-nucleotide repeats (18.33%), tetra-nucleotide repeats (2.41%), hexa-nucleotide repeats (0.55%), and penta-nucleotide repeats (0.35%). All SSRs and their primers are listed in Supplementary Table 2.

### Comparison with previous *L. vannamei* transcriptomes

**Figure 7:**
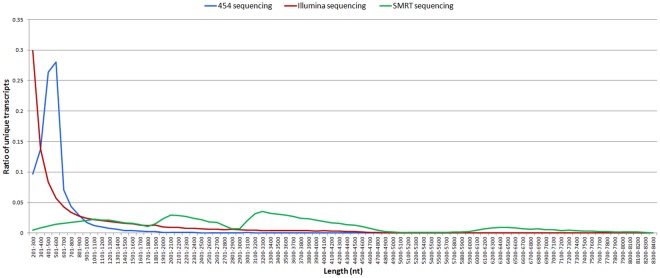
Lengths of unique transcripts in transcriptomes generated by SMRT sequencing (this study), 454 pyrosequencing^17^, and Illumina sequencing^18^.

**Figure 8:**
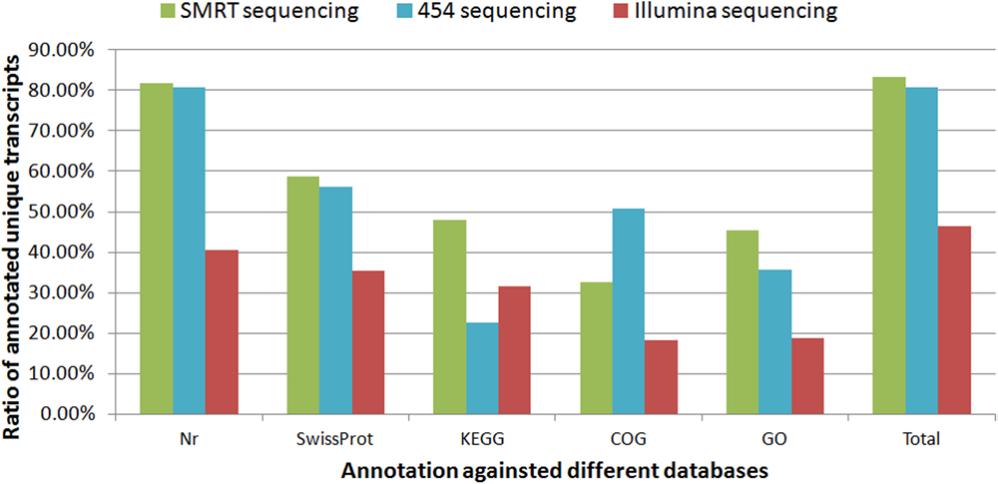
Successful functional annotations of unique transcripts in transcriptomes generated by SMRT sequencing (this study), 454 pyrosequencing^17^, and Illumina sequencing^18^.

Strikingly, most of the assembled unique transcripts generated by Illumina and 454 sequencing were <1000 bp in length, while the lengths of the SMRT assembled unique transcripts were much more evenly distributed, with a considerable proportion of assembled transcripts ~6000–8000 bp long (Fig. 7). With respect to transcript functional annotations, proportionally more SMRT-sequenced transcripts were annotated than either 454-pyrosequenced transcripts or Illumina-sequenced transcripts (Fig. 8).

## Discussion

Full-length cDNA sequences are useful for functional studies of important genes. However, full-length cDNA sequences can often only be generated by rapid amplification of cDNA ends (RACE), which is time consuming, labor intensive, expensive, and inefficient^29^. To date, very few full-length cDNA sequences have been reported for shrimp. Here, we used PacBio SMRT sequencing to obtain 51,367 high-quality unique full-length transcripts for *L. vannamei*. This large number of full-length cDNA sequences will greatly facilitate research projects using the shrimp transcriptome.

We compared several previously reported full-length cDNAs from *L. vannamei* with the corresponding full-length transcripts obtained in this study, including C-type lectin^30^, prophenoloxidase^31^, and ferritin^32^. We found the SMRT transcripts were essentially identical to the RACE cDNAs, with only minor differences at the 5′ and 3′ ends. These differences might have been due to differences in the primer sequences used by SMRT and RACE. Thus, our results suggested that SMRT sequencing is an effective method by which to obtain full-length cDNA sequences from the shrimp transcriptome.

Short-read sequencing (Illumina or 454) has been used to produce transcriptomes of some shrimp species, including *L. vannamei*^17,18,33–40^, *Fenneropenaeus merguiensis*^41,42^, *Macrobrachium rosenbergii*^43^, *Triops newberryi*^44^, *T. longicaudatus*^45^, *Pandalus latirostris*^46^, *Fenneropenaeus chinensis*^47^, *Palaemon serratus*^48^, and *Penaeus monodon*^49^. The average lengths of transcripts obtained in these studies were ~306–1,027 bp. Here, the average length of SMRT-sequenced transcripts was nearly 3 kb, far exceeding those of the previous studies. Our findings thus indicated that long transcripts in shrimp, from both coding and non-coding genes, might be more prevalent than previously estimated^33^.

Although SMRT sequencing produces longer reads than SGS methods, the SMRT raw data error rate is relatively high^50^. To correct these errors, it is possible to use the short reads generated by SGS as references^51,52^. Here, we used Illumina sequences to correct the SMRT reads. As 38.50% of the SMRT FLNC reads contained erroneous fragments (or single-nucleotide bases), our results indicated that error correction processing should be performed before further analysis of SMRT sequences.

LncRNAs are non-coding RNAs that are longer than 200 nucleotides long^53,54^. LncRNAs evolve rapidly, and are often species-specific in plants or animals^55^. An accumulating body of evidence has suggested that lncRNAs play essential roles in many important biological processes, such as translation, transcription, differentiation, splicing, immune responses, epigenetic regulation, and cell cycle control^54,56–59^. However, no lncRNAs in crustaceans have previously been reported. Here, we identified 3,958 novel lncRNAs in the *L. vannamei* shrimp transcriptome. These newly identified lncRNAs will be useful for several aspects of shrimp research, including epigenetics, immunology, and phylogenomics.

The SMRT transcriptome obtained here had a longer average transcript length than the transcripts obtained with SGS. Our results suggested that full-length transcripts were more easily annotated than shorter transcripts. Here, 81.72% of unique transcripts were annotated in the Nr database, as compared to 37.80%–73.08% in previously published *L. vannamei* transcriptomes produced with short-read sequencing^17,18,33,34^. This suggested that full-length transcripts were annotated more efficiently than the ESTs obtained by assembling short RNA-sequence reads.

## Materials and Methods

### Animal materials

Specific pathogen-free (SPF) white shrimp (*L. vannamei*) were obtained from the National and Guangxi Shrimp Genetic Breeding Center (Guangxi Province, China). We removed and pooled the hepatopancreases, gills, hearts, intestines, muscles, and stomachs of six shrimp. Pooled tissues were immediately stored in liquid nitrogen until RNA extraction.

### RNA extraction

Total RNA was extracted from the pooled tissues using TRIzol LS Reagent (Invitrogen, USA) following the manufacturer’s instructions, and genomic DNA was removed using DNase I (Invitrogen, USA). RNA purity (OD260/280), concentration, and absorption peak were measured using a NanoDrop 2000 (Thermo Scientific, USA). RNA quality was determined with a Bioanalyser 2100 (Agilent, USA). Only total RNAs with a RIN score >7 were used to construct cDNA libraries for SMRT sequencing.

### SMRT library construction, sequencing, and quality control

To construct full-length cDNAs, 10 μg of total RNA was reverse transcribed into cDNA using a SMARTer PCR cDNA Synthesis Kit (Takara, Japan), following the manufacturer’s protocols. Size fractionation and selection were performed using the BluePippin Size Selection System (Sage Science, USA). We prepared five SMRT libraries, each including fragments in one of five size groups: <1 kb, 1–2 kb, 2–3 kb, 3–6 kb, and >6 kb, following the PacBio protocol. Each library was sequenced in three SMRT cells on a PacBio RSII platform using C4 reagents and 3–4 h sequencing movies.

We used PacBio SMRT analysis software v2.3.0 (http://www.pacb.com/products-andservices/analytical-software/smrt-analysis/) to filter out low-quality polymerase reads (read-length <50 bp and read-score <0.75). ROIs were filtered from the sub-reads with the full pass threshold set to ≥0 and the predicted unique accuracy set to ≥0.75. We considered ROIs FLNC reads only if they possessed a 5′-cDNA primer, a 3′-cDNA primer, and a polyA tail preceding the 3′ primer. Then 5′- and 3′-cDNA primers and polyA tail were removed from FLNC according to the Pac-bio recommended procedure (https://github.com/PacificBiosciences/IsoSeq.3).

### Illumina library construction and sequencing

The Illumina libraries used to correct the FLNC reads were constructed with the Tru-Seq RNA sample Prep kit (Illumina, USA). Briefly, poly-(A) mRNA was isolated from total RNA using oligo (dT) magnetic beads and then fragmented into 200–700 bp pieces with fragmentation buffer. Double-stranded cDNAs were synthesized using a SuperScript double-stranded cDNA synthesis kit (Invitrogen, USA) with random hexamer primers (Illumina, USA), following the manufacturer’s instructions. Synthesized cDNAs were gen-purified and amplified with PCR. PCR products were sequenced on a single lane of an Illumina HiSeq. 2500 high-throughput sequencer. Raw sequencing reads were quality controlled to remove adaptor sequences, low-quality reads (reads where quality was ≤10% for >50% of all nucleotides), and read with many unknown nucleotides (>10%). Cleaned sequences were used for SMRT error correction.

### Quality filtering and error correction of PacBio reads

Nucleotide errors in the FLNC reads were corrected by comparison with the Illumina RNA sequences using Proovread v2.13.13 (https://github.com/BioInf-Wuerzburg/proovread) with parameter coverage set to 50^7,19^. Corrected FLNC reads were clustered into unique (non-redundant) transcripts using the ICE algorithm in the PacBio SMRT analysis software v2.3.0, with quiver polishing set to ≥0.99^55,60^. We used BUSCO v3.0 (http://busco.ezlab.org/)^20^ with the BUSCO arthropod dataset (http://busco.ezlab.org/v2/datasets/arthropoda_odb9.tar.gz) to evaluate the completeness of the *L. vannamei* transcriptome.

### Functional annotation of transcripts

We identified functional annotations matching each unique transcript by searching Nr, Swiss-Prot, COG, and KEGG using BlastX with an E-value cut-off of 10^−5^. Protein function was predicted based on the annotation of the most similar hit across all databases. The unique transcripts identified by BlastX were submitted to blast2GO v4.1 (http://www.blast2go.com)^61^ to assign GO categories. To identify the protein coding potential of each unique transcript, the ORFs within unique transcripts were predicted using TransDecoder v2.0.1 (https://transdecoder.github.io)^62^, with default parameters.

### Identification of lncRNAs

We identified unique transcripts without protein coding potential as candidate lncRNAs using four tools: CPC v1.0 (http://cpc.cbi.pku.edu.cn/)^24^, CNCI v2.0 (https://github.com/www-bioinfo-org/CNCI)^25^, CPAT v1.2 (http://lilab.research.bcm.edu/cpat/index.php)^26^, and Pfam (http://pfam.xfam.org/)^27^ with default parameters. We then predicted the ORFs of all candidate lncRNAs selected by at least one tool with EMBOSS getorf v6.1.0^63^; sequences containing ORFs > 100 bp long were discarded.

### Identification of SSRs

We used MISA v1.0 (http://pgrc.ipk-gatersleben.de/misa/)^64^ with default parameters to identify SSRs (mono- to penta-nucleotide repeats) in all corrected unique transcripts longer than 500 bp. SSR primers were designed using primer3^65^ with default parameters.

### Comparison with previously published *L. vannamei* transcriptomes

To evaluate SMRT sequencing performance, we compared the SMRT transcriptome constructed here to two previously published *L. vannamei* transcriptomes, one obtained using 454 sequencing^17^ and one obtained using Illumina sequencing^18^. First, we compared the distributions of transcript lengths among the three transcriptomes. Next, we compared the number of Nr, Swiss-Prot, KEGG, COG and GO hits among the transcriptomes (all functional annotations for each of the three transcriptomes were performed with an E-value cutoff of 10^−5^).

## Electronic supplementary material

Supplementary Table 1

Supplementary Table 2

## Data Availability

Raw PacBio sequencing reads are available at NCBI GenBank under the accession SRX3267788, SRX3267789, SRX3267790, SRX3267791, SRX3267792, SRX3267793, SRX3267794, SRX3267795, SRX3267796, SRX3267797, SRX3267798, SRX3267799, SRX3267800, and SRX3267801). Raw Illumina sequencing reads are available at NCBI GenBank under the accession SRX3527198 and SRX3527197. Candidate protein-coding transcripts are available at NCBI GenBank under the accession GGUK00000000. Candidate lncRNA sequences are available at NCBI GenBank under the accession GGUT00000000.
